# Supplementary material for: Cost of Recommended Diet (CoRD) and Its Affordability in Bangladesh
Source: Foods. 2023 Feb 13;12(4):790. doi: 10.3390/foods12040790 (PMC9956371; doi:10.3390/foods12040790)
Supplement: Supplementary file 1 [file foods-12-00790-s001.zip › foods-2078230-supplementary.pdf]

Supplementary Files

Table S1: List of two least-cost items under each food group

Food group	Name of divisions	Two least-expensive food items appearing under each food group in calculating the cost of a healthy diet
Cereals	Dhaka	Wheat, rice flakes
	Chittagong	Wheat, rice
	Mymensingh	Wheat, rice flakes
	Barisal	Wheat, rice
	Rajshahi	Wheat, rice
	Rangpur	Wheat, rice
	Sylhet	Wheat, rice
	Khulna	Wheat, rice
Pulses	Dhaka	Bengal gram, lentil
	Chittagong	Bengal gram, lentil
	Mymensingh	Bengal gram, black gram
	Barisal	Bengal gram, lentil
	Rajshahi	Bengal gram, lentil
	Rangpur	Bengal gram, lentil
	Sylhet	Bengal gram, lentil
	Khulna	Bengal gram, lentil
Non-leafy vegetables	Dhaka	Potato, radish
	Chittagong	Potato, radish
	Mymensingh	Bottle gourd, radish
	Barisal	Potato, radish

	Rajshahi	Bottle gourd, cabbage
	Rangpur	Radish, cabbage
	Sylhet	Potato, cabbage
	Khulna	Brinjal, radish
Leafy vegetables	Dhaka	Radish leaves, green amaranth leaves
	Chittagong	Jute leaves, radish leaves
	Mymensingh	Jute leaves, pumpkin leaves
	Barisal	Radish leaves, red amaranth leaves
	Rajshahi	Bottle gourd leaves, red amaranth leaves
	Rangpur	Water spinach, radish leaves
	Sylhet	Green amaranth leaves, Indian spinach
	Khulna	Radish leaves, spiny amaranth leaves
Fruits	Dhaka	Melon, carambola
	Chittagong	Banana, hog plum
	Mymensingh	Carambola, jujube
	Barisal	Carambola, melon
	Rajshahi	Carambola, papaya
	Rangpur	Carambola, banana
	Sylhet	Carambola, guava
	Khulna	Carambola, hog plum
Meat, fish and egg	Dhaka	Pool barb, egg
	Chittagong	Catfish, egg
	Mymensingh	Pool barb, egg
	Barisal	Pool barb, egg
	Rajshahi	Egg, silver carp

	Rangpur	Silver carp, egg
	Sylhet	Pool barb, egg
	Khulna	Egg, chicken meat
Milk and milk products	Dhaka	Cow’s milk, condensed milk
	Chittagong	Cow’s milk, condensed milk
	Mymensingh	Cow’s milk, condensed milk
	Barisal	Condensed milk, cow’s milk
	Rajshahi	Powdered milk, condensed milk
	Rangpur	Cow’s milk, condensed milk
	Sylhet	Cow’s milk, condensed milk
	Khulna	Condensed milk, powdered milk
Fats and oils	Dhaka	Soya oil, palm oil
	Chittagong	Palm oil, soya oil
	Mymensingh	Palm oil, soya oil
	Barisal	Soya oil, palm oil
	Rajshahi	Palm oil, coconut
	Rangpur	Palm oil, soya oil
	Sylhet	Soya oil, palm oil
	Khulna	Soya oil, palm oil
Sugar	Dhaka	Sugar, jaggery
	Chittagong	Sugar, jaggery
	Mymensingh	Sugar, jaggery
	Barisal	Sugar, jaggery
	Rajshahi	Sugar, jaggery
	Rangpur	Sugar, jaggery

Sylhet	Sugar, jaggery
Khulna	Sugar, jaggery

Table S2: District-wise estimates of the proportion of households unable to afford the CoRD

District	Proportion (95% CI)
Bagerhat	57.5 (53.9–61.1)
Bandarban	64.3 (60.8–67.9)
Barguna	29.8 (26.4–33.1)
Barisal	40.4 (36.8–44.0)
Bhola	16.7 (14.0–19.5)
Bogra	46.8 (43.1–50.5)
Brahmanbaria	11.6 (9.2–13.9)
Chandpur	25.8 (22.6–29.0)
Chapainawabganj	70.1 (66.8–73.5)
Chattagram	28.5 (25.2–31.8)
Chuadanga	67.1 (63.6–70.6)
Cumilla	14.3 (11.7–16.8)
Cox's Bazar	9.3 (7.2–11.4)
Dhaka	16.7 (14.0–19.4)
Dinajpur	67.5 (64.1–70.9)
Faridpur	18.6 (15.8–21.5)
Feni	21.5 (18.4–24.5)
Gaibandha	53.6 (49.9–57.2)
Gazipur	18.4 (15.5–21.2)

Gopalganj	44.6 (40.9–48.2)
Habiganj	44.8 (41.1–48.4)
Jamalpur	66.3 (62.8–69.7)
Jashore	64.8 (61.3–68.3)
Jhalakathi	33.4 (30.0–36.9)
Jhenaidah	68.1 (64.6–71.5)
Joypurhat	48.0 (44.3–51.6)
Khagrachhari	45.3 (41.7–49.0)
Khulna	68.4 (65.0–71.8)
Kishoreganj	59.7 (56.1–63.3)
Kurigram	75.4 (72.2–78.6)
Kushtia	57.2 (53.6–60.8)
Lakshmipur	25.0 (21.8–28.2)
Lalmonirhat	35.6 (32.1–39.1)
Madaripur	11.4 (9.1–13.7)
Magura	71.4 (68.1–74.7)
Manikganj	56.3 (52.6–59.9)
Moulvibazar	31.2 (27.8–34.6)
Meherpur	75.5 (72.3–78.6)
Munsiganj	22.1 (19.1–25.2)
Mymensingh	41.7 (38.1–45.3)
Naogaon	49.3 (45.6–53.0)
Narail	61.6 (58.1–65.2)
Narayanganj	10.1 (7.9–12.4)
Narsingdi	16.9 (14.1–19.7)

Natore	36.0 (32.5–39.5)
Netrakona	47.2 (43.6–50.9)
Nilphamari	34.5 (31.0–38.0)
Noakhali	21.8 (18.8–24.9)
Pabna	37.9 (34.4–41.5)
Panchagar	30.7 (27.3–34.1)
Patuakhali	37.8 (34.2–41.4)
Pirojpur	54.9 (51.2–58.5)
Rajbari	57.4 (53.8–61.1)
Rajshahi	39.5 (35.9–43.2)
Rangamati	13.4 (10.9–15.8)
Rangpur	49.7 (46.1–53.4)
Satkhira	64.6 (61.1–68.1)
Shariatpur	27.5 (24.2–30.8)
Sherpur	34.2 (30.7–37.6)
Sirajganj	37.2 (33.7–40.8)
Sunamganj	60.0 (54.6–63.6)
Sylhet	25.1 (21.9–28.3)
Tangail	36.2 (32.7–39.7)
Thakurgaon	33.4 (29.8–36.7)
